# Effect of empagliflozin on cardiac remodelling in South Asian and non-South Asian individuals: insights from the EMPA-HEART CardioLink-6 randomised clinical trial

**DOI:** 10.1186/s12872-023-03549-5

**Published:** 2023-11-15

**Authors:** William Barbour, Erika Wolff, Pankaj Puar, Makoto Hibino, Ehab Bakbak, Aishwarya Krishnaraj, Raj Verma, Meena Verma, Adrian Quan, Andrew T. Yan, Kim A. Connelly, Hwee Teoh, C. David Mazer, Subodh Verma

**Affiliations:** 1 Division of Cardiac Surgery, St. Michael’s Hospital of Unity Health Toronto, 30 Bond Street, Toronto, ON M5B 1W8 Canada; 2 Department of Physiology and Pharmacology, Western University, London, ON N6A 5C1 Canada; 3 School of Medicine, University College Cork, Cork, T12 K8AF Ireland; 4 Department of Pharmacology and Toxicology, University of Toronto, Toronto, ON M5S 1A8 Canada; 5 Faculty of Medicine, University of British Columbia, Vancouver, BC V6T 1Z3 Canada; 6 Division of Cardiothoracic Surgery, Emory University School of Medicine, Atlanta, GA 30322 USA; 7 School of Medicine, Royal College of Surgeons in Ireland, Dublin, D02 YN77 Ireland; 8 Division of Cardiology, St. Michael’s Hospital of Unity Health Toronto, Toronto, ON M5B 1W8 Canada; 9 Department of Medicine, University of Toronto, Toronto, ON M5S 1A8 Canada; 10 Department of Physiology, University of Toronto, Toronto, ON M5S 1A8 Canada; 11 Division of Endocrinology and Metabolism, St. Michael’s Hospital of Unity Health Toronto, 30 Bond Street, Toronto, ON M5B 1W8 Canada; 12 Department of Anesthesia, St. Michael’s Hospital of Unity Health Toronto, Toronto, ON M5B 1W8 Canada; 13 Department of Anesthesiology and Pain Medicine, University of Toronto, Toronto, ON M5G 1E2 Canada; 14 Department of Surgery, University of Toronto, Toronto, ON M5T 1P5 Canada

**Keywords:** Left ventricular mass regression, SGLT2 inhibition, South Asian ethnicity, Coronary artery disease, Type 2 diabetes mellitus

## Abstract

**Background:**

This exploratory sub-analysis of the EMPA-HEART CardioLink-6 trial examined whether the previously reported benefit of the sodium-glucose cotransporter 2 (SGLT2) inhibitor empagliflozin on left ventricular (LV) mass (LVM) regression differs between individuals of South Asian and non-South Asian ethnicity.

**Methods:**

EMPA-HEART CardioLink-6 was a double-blind, placebo-controlled clinical trial that randomised 97 individuals with type 2 diabetes mellitus (T2DM) and coronary artery disease (CAD) to either empagliflozin 10 mg daily or placebo for 6 months. LV parameters and function were assessed using cardiac magnetic resonance imaging. The 6-month changes in LVM and LV volumes, all indexed to baseline body surface area, for South Asian participants were compared to those for non-South Asian individuals.

**Results:**

Compared to the non-South Asian group, the South Asian sub-cohort comprised more males, was younger and had a lower median body mass index. The adjusted difference for LVMi change over 6 months was -4.3 g/m^2^ (95% confidence interval [CI], -7.5, -1.0; *P* = 0.042) for the South Asian group and -2.3 g/m^2^ (95% CI, -6.4, 1.9; *P* = 0.28) for the non-South Asian group (P_interaction_ = 0.45). There was no between-group difference for the adjusted differences in baseline body surface area-indexed LV volumes and LV ejection fraction.

**Conclusions:**

There was no meaningful difference in empagliflozin-associated LVM regression between South Asian and non-South Asian individuals living with T2DM and CAD in the EMPA-HEART CardioLink-6 trial.

**Trial registration:**

ClinicalTrials.gov Identifier: NCT02998970 (First posted on 21/12/ 2016).

## Background

One in four of the world’s population is of South Asian descent yet South Asian individuals account for approximately 60% of heart disease cases and half of cardiovascular (CV) deaths globally [1–4]. The excess CV risk is believed to be largely driven by the 2- to 3-fold higher prevalence of type 2 diabetes mellitus (T2DM) among individuals of South Asian descent relative to that among people of White European ancestry and other race/ethnicity [5–7]. The higher frequency of hypertension [8, 9], dyslipidaemia [10–13] and insulin resistance [14] as well as greater circulating levels of proinflammatory proteins [15, 16], body fat content and central adiposity [17] also contribute to the disproportionate CV risk among South Asian people.

Sodium-glucose cotransporter 2 (SGLT2) inhibitors, originally conceived as antihyperglycaemic agents, favourably lower the risk of CV events in people living with T2DM albeit with some heterogeneity [18–21] while risk of heart failure (HF) hospitalisations is more consistently reduced by the class regardless of diabetes status [18–25]. Like many contemporary clinical trials, participants of the large SGLT2 inhibitor cohorts were predominantly of White European ancestry individuals. Accordingly, the generalisability of the data and true impact of SGLT2 inhibition in people of non-White European descent remain unclear. Of note, meta-analyses using race/ethnicity-stratified data from SGLT2 inhibitor clinical outcome trials have yielded conflicting results with some suggesting that SGLT2 inhibitors may confer greater CV protection in Asian populations and others indicating that Asian and non-Asian individuals gain comparable CV benefits from this class of medications [26–28].

The EMPA-HEART (Effects of Empagliflozin on Cardiac Structure in Patients with Type 2 Diabetes) CardioLink-6 trial studied individuals with T2DM and coronary artery disease (CAD) and reported that empagliflozin assignment for 6 months led to significant reduction of left ventricular (LV) mass (LVM) indexed to body surface area (BSA) at baseline (LVMi) [29]. Inasmuch as LVM is deemed a predictor for CV events and CV mortality [30, 31], it has been suggested that the CV benefits of SGLT2 inhibition may be attributed in part to favourable LV remodelling. That approximately half of the EMPA-HEART CardioLink-6 cohort self-identified as being of South Asian descent offered the unique opportunity to gather insight on whether LVM regression by empagliflozin may differ between those of South Asian ethnicity and those who are not of South Asian ancestry.

## Methods

### Study design and participants

A detailed description of the EMPA-HEART CardioLink-6 study design, protocol and statistical analysis plan has been published [29]. In brief, EMPA-HEART CardioLink-6 was a double-blind, randomised, controlled, parallel-group trial (ClinicalTrials.gov Identifier: NCT02998970; first posted on 21/12/ 2016) that enrolled 97 individuals, aged 40–80 years, with an HbA1c between 6.5% and 10.0% and living with CAD, between 07/11/2016 and 05/04/2018 (inclusive) at St. Michael’s Hospital, Toronto, ON, Canada. Participants were centrally assigned (1:1) by the Applied Health Research Centre at the Li Ka Shing Knowledge Institute, with a concealed Web-based system and computer-generated random permuted blocks of sizes of 2 and 4, to receive either empagliflozin (10 mg/day *per os*, *n* = 49) or matching placebo (once daily *per os*, *n* = 48) for 6 months [29]. It was estimated that a total of 90 participants was necessary to provide 80% power to detect a between-group difference in LVM of 10 g from baseline to 6 months at a 2-sided α of 0.05. Demographic details were collected at the baseline visit—ethnicity was self-reported and participants were categorised as either South Asian or non-South Asian. Physical measurements, clinical history, laboratory values and pharmacotherapy details were documented at the scheduled clinic visits. Cardiac parameters and function were assessed with state-of-the-art cardiac magnetic resonance imaging (cMRI).

### Study outcomes

LVM, LV end-systolic volume (LVESV), LV end-diastolic volume (LVEDV) and LV ejection fraction (LVEF) were measured at the baseline and month 6 study visits. For statistical analyses, LVM, LVESV and LVEDV were also indexed against the participants’ baseline BSA. After the impact of placebo and empagliflozin assignment on the 6-month changes in LVEF as well as those of indexed and non-indexed LVM, LVESV and LVEDV were determined, the data were stratified into the South Asian and non-South Asian categories.

### Analysis

Normality of continuous variables was tested with the Skewness and Kurtosis test. Continuous variables are reported as median (interquartile range [IQR]); categorical data are expressed as frequencies and percentages. Continuous non-parametric variables were tested with the Mann–Whitney U test. Categorical variables were compared with a χ^2^ test followed by the Fisher's exact test where appropriate. The treatment effect on the 6-month changes in LV parameters are summarised as adjusted difference (95% confidence interval [CI]) and were assessed by ANCOVA, after adjusting for corresponding baseline values and following the intention-to-treat principle. A *P* value of < 0.05 was considered statistically significant. All statistical analyses were performed using Stata software (StataCorp. 2021. Stata Statistical Software: Release 17. College Station, TX: StataCorp LLC).

## Results

The baseline characteristics of the EMPA-HEART CardioLink-6 cohort are summarised in Table 1. Of the 97 participants randomised, 5 of those assigned to the empagliflozin group and 2 of whom were allocated to the placebo group were excluded from the final analysis due to missing outcome data, yielding a final sample size of 44 for the empagliflozin group and 46 for the placebo group. While over half (55%) of the participants self-identified as being of South-Asian descent, the remaining 44 self-identified as being of Caucasian (*n* = 32), East Asian (*n* = 5), African (*n* = 4), Aboriginal (*n* = 1) and other (*n* = 2) ancestries. Compared to the non-South-Asian group, the South Asian group had proportionally more men, a lower median age and body mass index as well as proportionally less individuals with a smoking history. Median T2DM duration was longer and median HbA1c higher in the South Asian group although proportionally less were insulin users. The two cohorts did not differ significantly with respect to any other baseline characteristics.

**Table 1:** Baseline characteristics of the EMPA-HEART CardioLink-6 cohort

	**South Asian**	**Non-South Asian**	***P* value**
	***n* = 53**	***n* = 44**	
Male	51 (96.2)	39 (88.6)	0.15
Age, years	59 (53, 68)	67 (63, 72)	0.003
BMI, kg/m^2^	26.0 (23.8, 28.1)	28.9 (25.1, 30.9)	0.003
HbA1c, %	8.0 (7.2, 8.6)	7.8 (7.4, 8.5)	0.63
Glucose (random), mmol/L	8.0 (6.6, 11.5)	9.3 (7.0, 13.6)	0.13
Systolic blood pressure, mmHg	126 (118, 139)	135 (128, 152)	0.014
Diastolic blood pressure, mmHg	74 (68, 80)	77 (70, 82)	0.22
Total cholesterol (random), mmol/L	3.2 (2.8, 3.6)	3.2 (2.8, 3.8)	0.76
LDL-cholesterol, mmol/L	1.3 (1.1, 1.8)	1.2 (1.0, 1.8)	0.74
HDL-cholesterol, mmol/L	1.0 (0.8, 1.2)	1.0 (0.9, 1.1)	0.87
Triglyceride, mmol/L	1.8 (1.3, 2.5)	1.9 (1.3, 2.1)	0.82
eGFR, mL/min per 1.73m^2^	85.9 (77.7, 97.9)	88.2 (77.2, 98.1)	0.93
Creatinine, mg/dL	0.9 (0.8, 1.0)	0.9 (0.8, 1.0)	0.52
Haemoglobin, g/dL	13.9 (12.6, 14.8)	14.1 (13.2, 15.1)	0.21
Haematocrit, %	0.42 (0.40, 0.44)	0.42 (0.40, 0.44)	0.41
NT-proBNP, pg/mL	97 (58, 190)	122 (55, 335)	0.19
**Clinical History**			
Duration of diabetes, years	10.0 (7.0, 16.0)	9.0 (3.0, 14.5)	0.062
Previous PCI	24 (45.3)	21 (47.7)	0.81
Previous CABG	33 (62.3)	22 (50.0)	0.22
Heart failure	4 (7.5)	2 (4.5)	0.69
Hypertension	50 (94.3)	38 (86.4)	0.29
Diabetic nephropathy	2 (3.8)	0 (0)	0.50
Stroke or TIA	7 (13.2)	7 (15.9)	0.78
Peripheral artery disease	2 (3.8)	3 (6.8)	0.66
Past or current smoker	17 (32.1)	25 (56.8)	0.014
**Medications**			
Metformin	50 (94.3)	41 (93.2)	1.00
Insulin	12 (22.6)	12 (27.3)	0.60
Statin	52 (98.1)	41 (93.2)	0.33
ACEi/ARB	45 (84.9)	36 (81.8)	0.79
Furosemide/Thiazide	5 (9.4)	10 (22.7)	0.093
ß blocker	41 (77.4)	36 (81.8)	0.62
Calcium channel blocker	12 (22.6)	9 (20.5)	1.00
ASA/P2Y_12_ inhibitor	46 (86.8)	35 (79.5)	0.41

Values are median (IQR) or n (%)

*ACEi* Angiotensin-converting enzyme inhibitor, *ARB* Angiotensin-receptor blocker, *ASA* Acetylsalicylic acid, *BMI* Body mass index, *CABG* Coronary artery bypass graft, *eGFR* Estimated glomerular filtration rate, *HbA1c* Glycated haemoglobin, *HDL* High-density lipoprotein, *LDL* Low-density lipoprotein, *NT-proBNP* N-terminal pro brain-type natriuretic peptide, *PCI* Percutaneous coronary intervention, *TIA* Transient ischaemic attack

The baseline LV parameters, as assessed by cMRI, are detailed in Table 2. Of note, LVM, LVESV and LVEDV, regardless of whether they were indexed to baseline BSA or not, were lower in the South Asian group relative to the non-South Asian cohort. There was no clinically meaningful difference in the LVEF values between the South Asian and non-South Asian participants.

**Table 2:** Baseline LV parameters of the EMPA-HEART CardioLink-6 cohort

	**South Asian**	**Non-South Asian**	***P* value**
	***n* = 53**	***n* = 44**	
Baseline LVM, g	104.6 (90.7, 116.2)	135.0 (112.3, 147.7)	< 0.001
Baseline LVMi, g/m^2^	55.5 (49.7, 61.8)	64.8 (56.7, 71.0)	< 0.001
Baseline LVESV, mL	48.2 (39.0, 59.8)	60.1 (46.5, 76.4)	0.003
Baseline LVESVi, mL/m^2^	25.3 (20.6, 30.9)	28.8 (22.7, 39.4)	0.085
Baseline LVEDV, mL	117.3 (101.1, 135.0)	150.1 (119.2, 164.6)	< 0.001
Baseline LVEDVi, mL/m^2^	63.4 (55.2, 71.7)	71.3 (61.5, 78.6)	0.016
Baseline LVEF, %	58.5 (52.6, 63.6)	58.1 (49.5, 63.6)	0.61

Values are median (IQR)

*LV* Left ventricular, *LVEDV(i)* LV end-diastolic volume (indexed to baseline body surface area), *LVEF* LV ejection fraction, *LVESV(i)* LV end-systolic volume (indexed to baseline body surface area), *LVM(i)* LV mass (indexed to baseline body surface area)

**Fig. 1:**
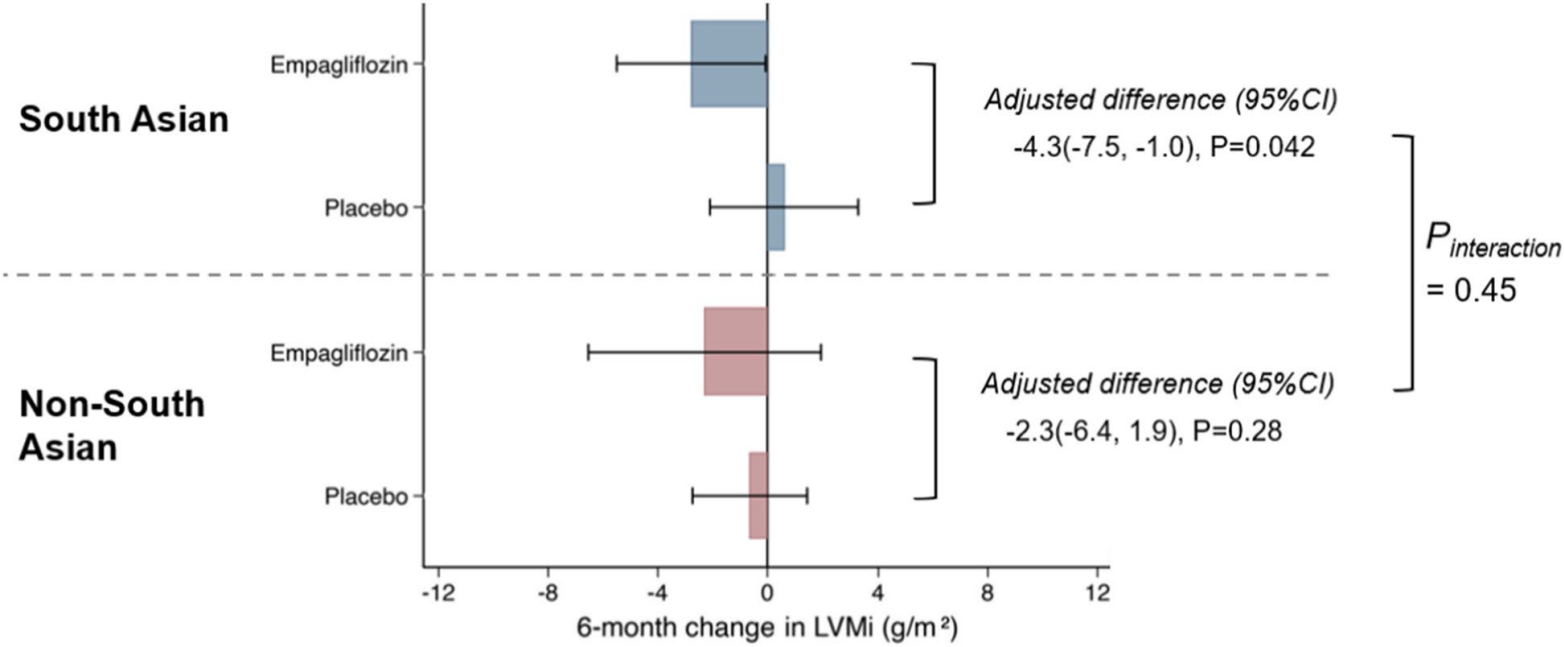
Effect of empagliflozin on LVMi in individuals with T2DM and CAD

**Fig. 2:**
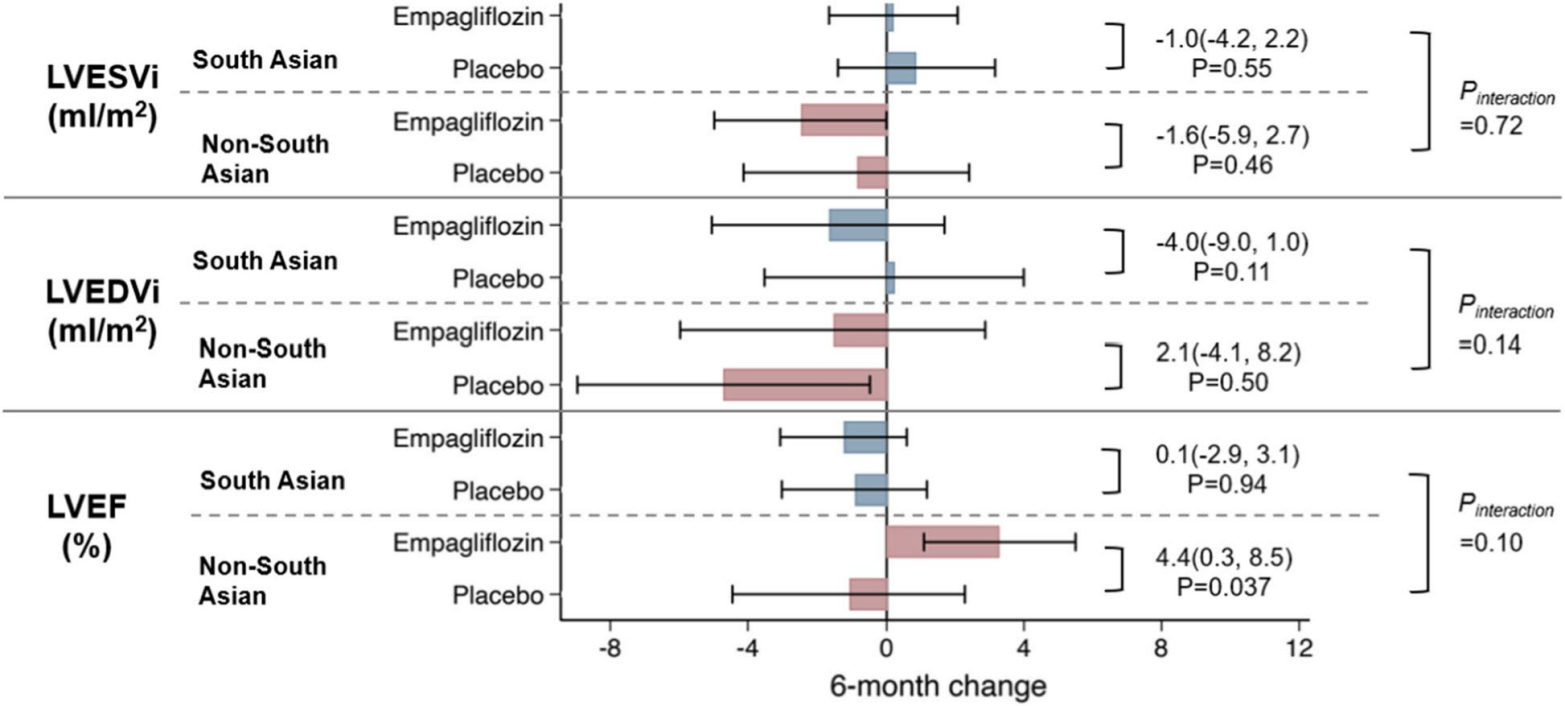
Effect of empagliflozin on LVESVi, LVEDVi and LVEF in individuals with T2DM and CAD

The treatment effect of empagliflozin and placebo assignment for 6 months on LVMi and LV volumes indexed to baseline BSA in both study groups are illustrated in Figs. 1 and 2. Figure 2 also shows the 6-month impact of empagliflozin and placebo treatment on LVEF in the South Asian and non-South Asian participants. The adjusted difference for LVMi change over 6 months in the South Asian group was -4.3 g/m^2^ while that for the non-South Asian group was 2.3 g/m^2^. This which yielded a P_interaction_ of 0.45. Of note, a regression analysis conducted in parallel revealed that regardless of ethnicity, baseline LVMi was the only factor that was associated with LVMi changes. The P_interaction_ values for the changes in LV volumes and LVEF over 6 months were not statistically significant.

The EMPA-HEART CardioLink-6 cohort was stratified into those of South Asian (*n* = 53) and non-South-Asian (*n* = 44) descent. Between-group data were analysed by ANCOVA, adjusting for baseline LVMi.

CAD, coronary artery disease; LVMi, left ventricular mass indexed to baseline body surface area; T2DM, type 2 diabetes mellitus.

The EMPA-HEART CardioLink-6 cohort was stratified into those of South Asian descent (*n* = 53) and non-South-Asian descent (*n* = 44). Between-group data were analysed by ANCOVA, adjusting for baseline LVMi.

CAD, coronary artery disease; LVEDVi, left ventricular end-diastolic volume indexed to baseline body surface area; LVEF, left ventricular ejection fraction; LVESVi, left ventricular end-systolic volume indexed to baseline body surface area; T2DM, type 2 diabetes mellitus.

## Discussion

This post hoc analysis of the EMPA-HEART CardioLink-6 trial revealed that while LVM regression occurred in both South Asian and non-South Asian participants following empagliflozin use for 6 months, there was no meaningful difference in empagliflozin-associated LVMi change between the two groups. Likewise, there was no meaningful between-group treatment-associated differences for LV volumes and LVEF.

Given that over half of the world’s population with pre-diabetes and diabetes are of Asian descent [32], it is perplexing that Asian individuals continue to be underrepresented in paradigm-changing diabetes and diabetes-related trials. Of note, diabetes is a primary driver of CV risk and despite robust evidence demonstrating that CV risk varies markedly across the Asian diasporas [33, 34], Asian participants are persistently aggregated in clinical trials. Importantly, not only do South Asian individuals carry a disproportionate brunt of atherosclerotic CV disease risk [35] but there is much heterogeneity within the South Asian population [36] underscoring the importance of collecting participant-level data. Indeed, the lack of granularity has perpetuated the paucity of information on how applicable trial findings from predominantly White European cohorts are to Asian individuals and for the matter, other non-White European populations.

Comprehensive findings from CV, kidney and HF trials with SGLT2 inhibitors since 2015 have persuaded diabetes, CV, kidney and HF clinical guideline writing committees to revise and update their recommendations more frequently than previous iterations. However, whether individuals of non-White European ancestry derive comparable benefits to those reported in the practice changing SGLT2 inhibitor trials remains unclear since most of the trial participants were of White European descent.

A meta-analysis that included five SGLT2 inhibitor trials – three CV outcome trials of T2DM cohorts and two with participants living with HF – suggested that participants of Asian descent reaped greater CV death reduction/HF hospitalisation benefits relative to the White European individuals [27]. In contrast, a more recent meta-analysis that considered four CV outcome trials of T2DM cohorts, two trials of participants with chronic kidney disease, and four trials that enrolled individuals living with HF, and consequently included more Asian participant data, reported no significant difference in SGLT2 inhibitor-derived CV death/HF hospitalisations between the Asian and White European populations [26]. Interestingly, both meta-analyses suggested that the SGLT2 inhibitor-associated benefits on major adverse cardiac events in Asian and White European individuals are comparable. Notwithstanding the different data sets, it must be acknowledged that the stratified and aggregated Asian data used by both meta-analyses were more than likely from participants with a wide range of risk factors who were on a multitude of background pharmacotherapies that may have had differing impact on the trial outcomes.

Aside from the EMPA-HEART CardioLink-6 investigators, five other groups have described cMRI-measured LV remodelling findings following SGLT2 inhibitor exposure. While all were randomised placebo-controlled trials, two followed less than 70 participants [37, 38] and two enrolled individuals without diabetes [39, 40]. The StUdies of empaGliflozin and its cArdiovascular, Renal and metabolic effects in patients with Diabetes Mellitus and Heart Failure (SUGAR-DM-HF) that enrolled individuals with pre-diabetes/T2DM and HF with reduced ejection fraction, reported declines in body surface area-indexed LV volumes but not LVMi 36 weeks after empagliflozin assignment [41]. Notably, the authors did not provide any race/ethnicity details on the study cohort although this is perhaps unsurprising given the cohort size of 105. Regardless, there are clearly contrasting results between the SUGAR-DM-HF and EMPA-HEART CardioLink-6 trials, and these may be attributed to several factors. First, 100% of the SUGAR-DM-HF cohort were living with HF whereas only 6% of the EMPA-HEART CardioLink-6 participants had a HF history. Second, SUGAR-DM-HF enrolled individuals with both prediabetes and T2DM while EMPA-HEART CardioLink-6 only included patients with an HbA1c between 6.5% and 10.0%. Third, SUGAR-DM-HF had a longer study duration (36 weeks) than EMPA-HEART CardioLink-6 (~ 26 weeks). It is therefore plausible that the different cardiac and metabolic milieus may have triggered different changes and responses to SGLT2 inhibition at the tissue, cellular and molecular levels. More specific to the current work, whether the same mechanisms are (de)activated by SGLT2 inhibitors and to the same extent in individuals of different race/ethnicity will require a much larger study.

This study has some strengths. First, the data were derived from a randomised controlled study that utilised gold standard cMRI methodologies to measure the LV parameters in a blinded fashion. Second, the sizes of the South Asian and non-South Asian groups were similar, and the baseline characteristics were relatively balanced. This work has some limitations. First, the overall sample size was small and the study duration short, thus limiting the generalisability of the findings. Second, the ethnic heterogeneity within the non-South Asian arm likely culminated in an imbalanced distribution of CV risk factors; likewise, since specific South Asian ancestry data were not collected, it is unknown how diverse the CV risk profiles among the South Asian patients was.

## Conclusions

In the EMPA-HEART CardioLink-6 trial, there did not appear to be any meaningful difference in empagliflozin-associated LVM regression between the South Asian and non-South Asian participants.

## Data Availability

The dataset may be available from the corresponding author upon reasonable request.

## Abbreviations

BSA: Body surface area
CAD: Coronary artery disease
CI: Confidence interval
CV: Cardiovascular
HF: Heart failure
IQR: Interquartile range
LV: Left ventricular
LVEDV: Left ventricular end-diastolic volume
LVEF: Left ventricular ejection fraction
LVESV: Left ventricular end-systolic volume
LVM: Left ventricular mass
LVMi: Left ventricular mass indexed to body surface area
SGLT2: Sodium-glucose cotransporter 2
T2DM: Type 2 diabetes mellitus
